# HECT E3 Ligases: A Tale With Multiple Facets

**DOI:** 10.3389/fphys.2019.00370

**Published:** 2019-04-03

**Authors:** Janine Weber, Simona Polo, Elena Maspero

**Affiliations:** ^1^Fondazione Istituto FIRC di Oncologia Molecolare, Milan, Italy; ^2^Dipartimento di Oncologia ed Emato-Oncologia, Università degli Studi di Milano, Milan, Italy

**Keywords:** ubiquitin, E3 ligase, cancer, inhibitor, HECT regulations

## Abstract

Ubiquitination plays a pivotal role in several cellular processes and is critical for protein degradation and signaling. E3 ubiquitin ligases are the matchmakers in the ubiquitination cascade, responsible for substrate recognition. In order to achieve selectivity and specificity on their substrates, HECT E3 enzymes are tightly regulated and exert their function in a spatially and temporally controlled fashion in the cells. These characteristics made HECT E3s intriguing targets in drug discovery in the context of cancer biology.

## HECT E3 Ligases

Post-translational modification of proteins by the addition of a ubiquitin (Ub) moiety can induce alteration in protein stability, function, activity, localization and interaction (Komander and Rape, 2012). This tightly regulated process (reviewed in Oh et al., 2018) requires the sequential action of a cascade of three enzymes: the Ub-activating enzyme (E1), Ub-conjugating enzymes (E2s), and Ub ligases (E3s). E3 ligases are the matchmakers of the enzymatic cascade, as they are capable of conferring a high degree of specificity and selectivity toward target substrates in cells. The human proteome codifies for more than 600 E3s (Li et al., 2008; Berndsen and Wolberger, 2014) that can be divided into three classes: the largest class is the RING (Really Interesting New Gene/U-box)-type E3s with about 600 members (reviewed in Metzger et al., 2014), followed by the HECT (Homologous to E6AP C-Terminus)-type E3s with about 28 members (reviewed in Rotin and Kumar, 2009 and Sluimer and Distel, 2018) and the RBR (RING between RING)-type E3s with about 14 members (reviewed in Dove and Klevit, 2017 and Reiter and Klevit, 2018). Whereas the RING E3 ligases function as allosteric activators of the E2 and scaffolds that bring the latter in close proximity to the substrate, the HECT and RBR E3 ligases catalyze substrate ubiquitination in a two-step reaction: in the first step, they accept the activated Ub from the E2 in a transthiolation reaction on their catalytic cysteine, and in the second step, the Ub moiety is transferred to a lysine on the target substrate.

In this review, we focus on the HECT-containing E3 ligases. Invariably at their C-terminus, all HECT E3s present the catalytic HECT domain, composed of a bulkier N-terminal lobe (N-lobe) that contains the E2 binding domain, and a C-terminal lobe (C-lobe) carrying the catalytic cysteine. The two lobes are connected by a flexible hinge region that allows the C-lobe to move around in order to facilitate the Ub transfer from the E2 to the E3 (Huang et al., 1999; Verdecia et al., 2003; Kamadurai et al., 2009).

According to the domain organization present in the N-terminal part of the proteins, the HECT E3s can be subdivided into three main families (Table 1; Rotin and Kumar, 2009; Scheffner and Kumar, 2014; Sluimer and Distel, 2018). The best characterized family is the NEDD4 family, consisting of nine human members: ITCH, SMURF1, SMURF2, WWP1, WWP2, NEDD4, NEDD4-2, HECW1, and HECW2. The NEDD4 members share similar domain structure and consist of a membrane/lipid-binding C2 domain, two to four WW domains for substrate recognition and a C-terminal HECT domain (Fajner et al., 2017). The second class, the HERC family, is characterized by one or more regulators of chromatin condensation 1 (RCC)-like domains (RLD), which serve as a guanine nucleotide exchange factor (GEF) for the small GTPase in membrane trafficking processes (Sanchez-Tena et al., 2016). This family consists of six members that can be subdivided into four ‘small’ and two ‘large’ HERCs, where the latter, HERC1 and HERC2, are the largest HECT E3s with about 5000 residues. The remaining 13 HECTs do not share specific domains at the N-terminus and, for this reason, are classified as “other” HECT ligases (Scheffner and Kumar, 2014).

**Table 1:** Overview of the human HECT E3 ligase subfamilies with the respective members, including their domain organization.

Subfamily	Domains	Members
NEDD4	C2, WW (4x), HECT	NEDD4, NEDD4-2, ITCH, WWP1, WWP2
	C2, WW (3x), HECT	SMURF2
	C2, WW (2x), HECT	SMURF1, HECW1, HECW2
HERC	RLD (2x), SPRY, WD40, HECT	HERC1
	RLD (3x), Cytb5, MIB, ZnF, DOC, HECT	HERC2
	RLD, HECT	HERC3, HERC4, HERC5, HERC6
“Other” HECT	AZUL, HECT	E6AP
	ARM, UBA, WWE, BH3, UBM, HECT	HUWE1
	ANK, HECT	HACE1
	ARM (2x), WWE, HECT	TRIP12
	UBA, ZnF, PABC, HECT	UBR5
	IQ, HECT	UBE3B, UBE3C
	ANK, SUN, MIB, HECT	HECTD1
	HECT	HECTD2, HECTD4
	DOC, HECT	HECTD3
	PHD/RING, HECT	G2E3
	Filamin, HECT	AREL1


*Protein domains were predicted by the InterPro server (Finn et al., 2017). HECT E3 ligases are grouped into three subfamilies, NEDD4, HERC, and “other” HECT E3 ligases based on their domain architecture N-terminal to the HECT domain. Domain abbreviations used are as follows: *C2*, C2 domain (Ca^*2*+^-binding domain); *WW*, WW domain; *HECT*, homologous to E6AP C-terminus; *RLD*, Regulator of Chromosome Condensation 1 repeat-like domain; *SPRY*, B30.2/SPRY domain; *WD40*, W-D repeat domain; *Cytb5*, cytochrome b5-like heme/steroid-binding domain; *DOC*, APC10/DOC domain; *MIB*, MIB-HERC2 domain; *ZnF*, Zinc finger domain; *AZUL*, amino-terminal Zn-binding domain of UBE3A ligase; *ARM*, Armadillo repeat-containing domain; *UBA*, ubiquitin-associated domain; *WWE*, WWE domain; *BH3*, Bcl-2 homology 3 domain; *UBM*, ubiquitin-binding motif; *ANK*, Ankyrin repeat-containing domain; *PABC*, polyadenylate-binding protein C-terminal domain; *IQ*, IQ motif/EF-hand binding site; *SUN*, SAD1/UNC domain; *PHD*, PHD-type zinc finger; *Filamin*, filamin/ABP280 repeat-like domain*.

## Regulation of HECT E3 Ligase Activity

The activity of HECT E3s is tightly regulated in terms of chain specificity (mono- or poly-ubiquitination and Ub chain linkage), interaction with the E2 and recognition of the substrate.

The ability to build linkage-specific poly-Ub chains appears to be an intrinsic feature of the HECT enzymes, as they are able to generate distinct Ub chains regardless of the paired E2 enzymes. NEDD4 family members primarily synthesize K63 chains (Kim and Huibregtse, 2009; Maspero et al., 2013; Kristariyanto et al., 2015), while E6AP is a K48-specific enzyme (Wang and Pickart, 2005; Kim and Huibregtse, 2009) and HUWE1 generates K6-, K11-, and K48- linked poly-Ub chains (Jackl et al., 2018). In most of the cases, the detailed mechanism through which they assemble specific poly-Ub chains remains unknown. In the case of NEDD4, the presence of a non-covalent Ub-binding site, called the Ub exosite, in the N-lobe appears to be required for enzyme processivity, possibly by stabilizing and orienting the distal end of growing Ub chains on the substrate (Maspero et al., 2011, 2013).

Precise control of E3 ligase activity is needed to ensure that their functions are restricted until required. Several HECT E3s are kept in a catalytically inactive state by intramolecular interactions between the N-terminal region (either the C2 or the linker region between the WW domains) and the C-terminal HECT domain (Wiesner et al., 2007; Mari et al., 2014; Riling et al., 2015; Chen et al., 2017; Zhu et al., 2017). For other E3s, such as E6AP and HUWE1, the mechanism is different but always requires intermolecular interactions. The crystal structure of the free HECT domain of E6AP suggests that it forms a trimer (Huang et al., 1999) and that the trimeric state activates the E3 ligase (Ronchi et al., 2014). In contrast, the C-terminal region of HUWE1 is maintained in an inactive conformation by homo-dimerization that occurs at the HECT domain. The engagement of the dimerization region by an activation segment located at the N-terminal of the protein seems to relieve this inhibitory mechanism (Sander et al., 2017).

A third level of regulation is represented by adaptor proteins that can modulate both the E2–E3 interaction and the interaction with the substrate. An example of the former is represented by SMAD7. SMURF1 and SMURF2 have low binding affinities for the E2-conjugating enzyme UbcH7, providing a point of control for regulating the Ub ligase activity through the action of auxiliary proteins. Indeed, SMAD7, functioning as a bridge between the E2 and E3, stabilizes an active complex and promotes, thus, the ligase activity (Ogunjimi et al., 2005). In other cases, adaptor proteins may regulate the E3 ligase by promoting its engagement with the substrate. The most famous example is represented by the adaptor protein E6 that binds to a LxxLL motif of the E6AP HECT ligase and forms, together with E6AP, a binding surface for the p53 protein. Consequently, p53 becomes K48-poly-ubiquitinated and degraded by the 26S proteasome (Huibregtse et al., 1993; Martinez-Zapien et al., 2016). NEDD4 family E3s usually recruit substrates via the WW domains that serve as direct binding sites for PPxY motifs present on the targets (Persaud et al., 2009). In this case, cooperation with auxiliary proteins confers the ability to cope with a larger number of substrates. Indeed, in the last decade, proteins such as ARTs in yeast and ARRDCs in mammals were found to modulate the ubiquitination of PY-negative substrates (Lin et al., 2008; Polo and Di Fiore, 2008; Mund and Pelham, 2009; Han et al., 2013). Other adaptor proteins which contribute to NEDD4 family members regulation are NDFIP1 and NDFIP2, transmembrane proteins that localize to Golgi, endosomes, and multivesicular bodies (Harvey et al., 2002; Shearwin-Whyatt et al., 2006). Through their cytoplasmic PY motifs they allow the association of NEDD4 family members to their specific substrates [e.g., DMT1 (Foot et al., 2008), ENaC (Konstas et al., 2002) the water channel AQP2 (Trimpert et al., 2017)] and directly modulate the activity of these E3s (Mund and Pelham, 2009, 2010).

Notably, HECTs themselves can function as adaptors for other conjugating enzymes as in the case of HERC2 whose binding to the N-terminal domain of E6AP increases the catalytic activity of E6AP (Kuhnle et al., 2011).

Finally, the catalytic activity of the HECT enzymes is often spatially and temporally regulated by post-translational modifications. Activating modifications can contribute to the release of auto-inhibiting conformational states of the E3s. For example, the phosphorylation of ITCH on the three residues of the proline-rich region releases the auto-inhibitory state generated by the binding of the C2 and the first WW domain to the HECT domain (Gallagher et al., 2006). Likewise, FGFR and EGFR activate NEDD4 by inducing a Src-dependent phosphorylation of specific tyrosine residues in the C2 and HECT domains, opening thus the closed conformation (Persaud et al., 2014); a mechanism that seems to be in place also for NEDD4-2 (Grimsey et al., 2018). With an opposite behavior, phosphorylation of a specific residue in the HECT domain of E6AP by the kinase c-Abl disrupts the trimeric state and therefore inhibits the ligase (Chan et al., 2013).

## HECT E3 Ligases and Their Undefined Role in Tumorigenesis

Ubiquitin ligases regulate a wide range of cellular processes and are involved in many human pathologies. Abnormal expression or dysfunction of HECT E3s have been shown in many different cancers. The current knowledge often suggests a dual role for these ligases in tumorigenesis, which might depend on the tissue context and/or additional events that affect their activity. Here, we will review the recent literature on E6AP, NEDD4, and HUWE1, and highlight excellent reviews for additional reading (Bernassola et al., 2008; Rotin and Kumar, 2009; Scheffner and Kumar, 2014; Zou et al., 2015; Wang et al., 2017; Kao et al., 2018).

The classical example of an HECT associated with cancer is E6AP. Since its discovery in 1993, it was evident that E6AP drives human papilloma virus (HPV)-induced cervical carcinogenesis, exerting its activity toward the tumor suppressor p53 through its association with the viral protein E6. E6 is an adaptor protein of the HPV and it is capable of binding to the N-terminal of EA6P and the DNA-binding domain of p53 (Huibregtse et al., 1993; Scheffner et al., 1993; Beaudenon and Huibregtse, 2008), acting as an allosteric activator of E6AP (Mortensen et al., 2015), similarly to HERC2 that binds to the same region (Kuhnle et al., 2011). In addition to HPV-induced cancer, E6AP drives cancer progression in B-cell lymphoma where it degrades PML, allowing the tumor cells to bypass PML-induced senescence (Wolyniec et al., 2012). While E6AP appears to have a pro-oncogenic function, a few papers support a tumor-suppressive function for E6AP in breast and prostate cancers (Srinivasan and Nawaz, 2011; Levav-Cohen et al., 2012; Ramamoorthy et al., 2012; Mansour et al., 2016) and in non-small cell lung cancer where depletion of E6AP contributes to a decreased expression of the INK4/ARF locus (Gamell et al., 2017).

NEDD4 and NEDD4-2 E3s are instead modulators of endocytosis of several membrane proteins such as growth factor receptors [EGFR (Katz et al., 2002) and IGFR (Vecchione et al., 2003; Cao et al., 2008)] and ion channels [ENaC (Staub et al., 1996; Harvey et al., 2001), Na_v_s (Fotia et al., 2004), and KCNQs (Ekberg et al., 2007; Jespersen et al., 2007)] therefore they are important players in the maintenance of cellular homeostasis. Mutations at the C-terminal of ENaC subunits that abrogate the interaction with NEDD4-2 are the cause of Liddle’s syndrome, an autosomal dominant disorder with severe sodium retention and hypertension (Staub et al., 1996).

Overexpression of NEDD4 has been reported in several cancer types and downregulation of NEDD4 appears to reduce proliferation, migration and invasion of cancer cells (reviewed in Zou et al., 2015). The relevance of NEDD4 in the tumorigenic process was initially associated with the identification of the tumor suppressor PTEN as a NEDD4 substrate (Trotman et al., 2007; Wang et al., 2007; Kim et al., 2008; Amodio et al., 2010). Later observations linked RAS activation to NEDD4 overexpression and subsequent PTEN degradation in human colorectal cancer (Zeng et al., 2014). However, studies conducted in NEDD4 knock out (KO) mice showed that PTEN stability was not affected by the E3 ligase deficiency (Fouladkou et al., 2008), while others showed that overexpression of NEDD4 in colorectal cancers promotes cancer cell growth independently of PTEN and PI3K/AKT signaling, arguing that NEDD4-mediated regulation of PTEN is microenvironment and/or cell-type specific, and that other yet-unknown substrates are implicated in the process (Eide et al., 2013). While this latter remains an intriguing hypothesis, it is interesting to note that *in vivo* NEDD4 is reported to degrade many of its substrates, while *in vitro* its activity is clearly K63-specific (Kim and Huibregtse, 2009; Maspero et al., 2013). A possible explanation for this behavior resides in the involvement of adaptor proteins that could influence the specific type of Ub chains catalyzed by E3s [e.g., NUMB (Shao et al., 2017)] or deubiquitinases that may edit the Ub chains.

A last case study is represented by HUWE1, which is also frequently overexpressed in tumors (Chen et al., 2005; Confalonieri et al., 2009; Myant et al., 2017). Again, HUWE1 has been associated with both pro-oncogenic and tumor suppressor functions since it is responsible for K48-mediated degradation of a great variety of substrates ranging from the oncoprotein MYC (Zhao et al., 2008; Inoue et al., 2013; Myant et al., 2017) to the anti-apoptotic protein MCL1, (Zhong et al., 2005) to the tumor suppressor p53 (Chen et al., 2005) and BRCA1 (Wang et al., 2014). Particularly controversial is the role of HUWE1 in the regulation of MYC. On the one hand, HUWE1 is able to enhance tumor cell proliferation by K63-poly ubiquitination and activation of the transcription regulator MYC (Adhikary et al., 2005), on the other hand, depletion (Inoue et al., 2013) or mutation (Myant et al., 2017) of HUWE1 lead to increased MYC levels, thereby promoting skin and colon tumorigenesis. Clearly, a precise understanding of HUWE1 function in the various cancers relies heavily on the identification of its direct substrates and the type of Ub modification occurring to them.

## Target Sites and Specificity of HECT E3 Ligase Inhibitors

**FIGURE 1:**
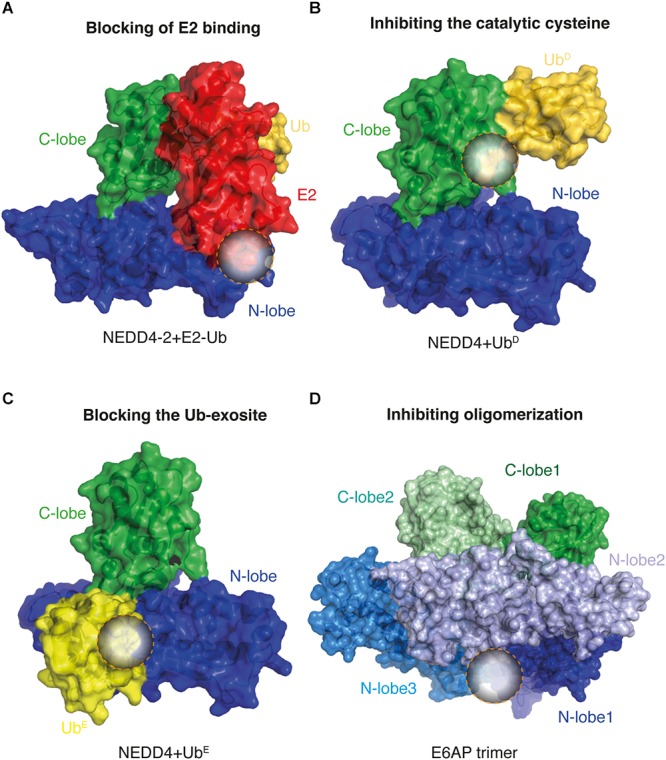
Predicted inhibitory targets for HECT E3s activity. Surface representations of known crystal structures of HECT E3s. In circles are highlighted the possible binding sites for small molecules. **(A)** Blocking the E2 binding: HECT^NEDD4-2^ in complex with Ub (gold)-loaded E2 (red; PDB 3JVZ). **(B)** Inhibition of the catalytic cysteine: Ub (Ub^D^, gold)-loaded HECT^NEDD4^ complex; PDB 4BBN. **(C)** Blocking the Ub exosite: HECT^NEDD4^ in complex with Ub bound to the exosite (Ub^E^, yellow; PDB 4BBN). **(D)** Inhibition of the oligomerization: HECT^E6AP^ trimeric complex (1D5F). C-lobes and N-lobes are depicted in green and blue, respectively.

As previously described, the regulatory mechanisms of HECT E3s are quite diverse and, therefore, provide a promising opportunity for drug discovery (Chen et al., 2018). Based on the actual knowledge, we can imagine different ways to inhibit their activity, namely: (i) by blocking the binding of the E2 enzymes or adaptor proteins; (ii) by tackling the catalytic cysteine of the enzymes; (iii) by targeting specific regulatory surfaces such as the Ub exosite; (iv) by impairing substrate recognition; and (v) by modulating the oligomeric state (Figure 1).

Molecules that block the HECT-E2 binding were found by Mund et al. (2014). By using a phage library, the authors isolated and modified bicyclic peptides that specifically bind to the HECT domains of SMURF2, NEDD4-1, WWP1, and HUWE1, competing with the E2 binding. Further improvement of the most promising peptide generated Heclin (HECT ligase inhibitor), a reversible inhibitor with a low micromolar affinity that, however, did not inhibit the E2 binding of the HECTs but rather caused a conformational change that renders their catalytic cysteine more susceptible to oxidation.

With the idea of identifying covalent modifiers of the catalytic cysteine of NEDD4, Kathman et al. (2015) found compounds that selectively react with a non-catalytic cysteine present in the Ub exosite of NEDD4 and NEDD4-2. Interestingly, no inhibition was observed for the NEDD4 family member WWP1 that also contains a cysteine in close proximity to the one seen in NEDD4, or for E6AP that does not contain a cysteine in this region (Kathman et al., 2015). Another compound that may bind to the Ub exosite of the HECT domain is I3C (1H-indol-3-yl-carbinol), a phytochemical found in cruciferous vegetables that has an antiproliferative effect in cancers (Ahmad et al., 2010). I3C was found to interact with NEDD4 *in vitro* at micromolar concentrations (Adhikary et al., 2005). Through *in silico* binding simulations between I3C and the NEDD4 crystal structure, I3C was predicted to bind to the hydrophobic pocket of the N-lobe near the Ub exosite. In a follow-up study, Quirit et al. (2017) overcame the low binding affinity of I3C by screening a small library of *N*-benzyl or *N*-phenyl I3C analogs and identified 1-benzyl-indole-3-carbinol (1-benzyl-I3C) as a more potent inhibitor. However, the binding mode and the specificity of this compound has not been experimentally validated.

A recent approach suggests the use of specific Ub mutants identified by phage display (Ub variant, UbV) to modulate HECT catalysis (Zhang et al., 2016). The screen performed against 19 of the 28 human HECT enzymes lead to the identification of variants that are capable to bind the N-lobe exosite but also the N-lobe surface involved in the interaction with the E2 (Zhang et al., 2016). Binding of these variants promote inhibition or activation depending on the E3 tested and the type of modifications present in the UbV underlying the complexity of the catalytic mechanism in place. While a generalization of the process is impossible, these reagents appear to be interesting tools for further studies.

A few molecules have been found to inhibit the HECTs, impairing substrate binding. An *in silico* screening of the hydrophobic pocket of the WW domains of SMURF1 led to the identification of compounds that possess features similar to the PPXY motif. These compounds bind the ligase and block SMAD1 ubiquitination, possibly disrupting the WW domain:SMAD1 interaction (Okada et al., 2009; Kato et al., 2011; Cao et al., 2014). However, affinity, binding mode and selectivity remain to be tested.

For many small molecules and inhibitors, the binding sites, the mechanism or the specificity have not been determined. By a high-throughput screening of small molecules, Eilers and co-workers identified two compounds that selectively inhibit the enzyme activity of HUWE1, as seen by a reduced substrate ubiquitination. Both compounds were found to inhibit the ligase activity with IC_50_ values in the low micromolar range, leaving NEDD4 family members or the E1 and E2 enzymes unaffected. The compounds reduced the growth of colorectal cancer cells, but not that of HUWE1-depleted or normal epithelial cells of the colon (Peter et al., 2014). However, how these compounds work on HUWE1 remains unknown (Peter et al., 2014). With a similar approach, Rossi and co-workers identified several putative ITCH inhibitors, including Clomipramine, an FDA-approved drug that is used in the clinic to treat psychiatric disorders. Clomipramine and its analogs specifically block the HECT catalytic activity of the NEDD4 family member ITCH but not that of the RING ligase Ring1B (Rossi et al., 2014). The authors clarified the mode of action of this class of drugs, showing that it specifically inhibits the transthioesterification reaction (the transfer of Ub from the E2 to the HECT domain), implying some common features at the level of the HECT members (Rossi et al., 2014).

## Concluding Remarks

Although tackling the ubiquitination system rather than the proteasome seems to be a promising avenue for therapeutic drug discovery, targeting HECT E3s to manipulate their activity is challenging for several reasons. First of all, we still lack the complete picture of their ubiquitome and their mechanism of action. Which substrates do HECT E3s ubiquitinate? What impact does ubiquitination have on their function proteolytic or non-proteolytic? How are these substrates recognized and how is their ubiquitination regulated in time and space and in different cellular conditions? What are the mechanisms the different HECT E3s apply to ubiquitinate their targets? So far, we only have a few answers for a small number of ligases and substrates due to the fact that ubiquitination is a dynamic and highly regulated process, and that the interaction with substrates is often transient with a low binding affinity. Besides the PPxY motif that is recognized by the WW domains of NEDD4 family members, no other substrate binding motif is known. An additional challenge is represented by redundancy. While E3s target multiple substrates, a specific substrate may be modulated by several E3s, depending also on the cell context. The high conservation of the HECT domain within the HECT family makes it a difficult target for which to develop specific inhibitory compounds. Finally, most of the HECTs act as both tumor suppressors and oncogenes, and more information is needed in order to find specific and effective compounds. Thus, acquiring more insights into the structural composition and the ubiquitination mechanism used by the different HECT E3s is of paramount importance in order to open new avenues for therapeutic interventions.

## Author Contributions

JW, EM, and SP conceptualized and wrote this review. JW prepared the figure. All authors approved the final version of the manuscript and agreed to be accountable for the content of the work.

## Conflict of Interest Statement

The authors declare that the research was conducted in the absence of any commercial or financial relationships that could be construed as a potential conflict of interest.
